# Short-Term Intake of Yellowstripe Scad versus Salmon Did Not Induce Similar Effects on Lipid Profile and Inflammatory Markers among Healthy Overweight Adults despite Their Comparable EPA+DHA Content

**DOI:** 10.3390/nu13103524

**Published:** 2021-10-08

**Authors:** Wei Lin Chang, Azrina Azlan, Sabariah Md Noor, Irmi Zarina Ismail, Su Peng Loh

**Affiliations:** 1 Department of Nutrition, Faculty of Medicine and Health Sciences, Universiti Putra Malaysia, Serdang 43400, Selangor, Malaysia; chang.weilin@outlook.com (W.L.C.); azrinaaz@upm.edu.my (A.A.); 2 Department of Pathology, Faculty of Medicine and Health Sciences, Universiti Putra Malaysia, Serdang 43400, Selangor, Malaysia; md_sabariah@upm.edu.my; 3 Department of Family Medicine, Faculty of Medicine and Health Sciences, Universiti Putra Malaysia, Serdang 43400, Selangor, Malaysia; irmiismail@upm.edu.my; 4 Department of Nutrition, Faculty of Public Health, Universitas Airlangga, Jl. Mulyorejo Kampus C, Surabaya 60115, Indonesia

**Keywords:** dietary fish, yellowstripe scad, salmon, omega-3 fatty acids, eicosapentaenoic acid, docosahexaenoic acid, lipid profile, inflammatory markers, randomized crossover trial

## Abstract

Yellowstripe scad (YSS) have comparable eicosapentaenoic acid and docosahexaenoic acid (EPA+DHA) content to salmon. We aimed to compare the effects of YSS and salmon on lipid profile and inflammatory markers. A randomized crossover trial with two diet periods was conducted among healthy overweight (with BMI 23.0–27.4 kg/m^2^) Malaysian adults aged 21–55 years. Steamed whole YSS fish (≈385 g whole fish/day) or salmon fillets (≈246 g fillet/day) were given for eight weeks (3 days per week), retaining approximately 1000 mg EPA+DHA per day. Diets were switched after an 8-week washout period. Fasting blood samples were collected before and after each diet period. A total of 49 subjects participated in the intervention (35% male and 65% female; mean age 29 (7) years). YSS did not induce any significant changes in outcome measures. However, the consumption of salmon as compared with YSS was associated with reduction in triglycerides (between-group difference: −0.09 mmol/1, *p* = 0.01), VLDL-cholesterol (between-group difference: −0.04 mmol/1, *p* = 0.01), atherogenic index of plasma (between-group difference: −0.05 mmol/1, *p* = 0.006), and IL-6 (between-group difference: −0.01 pg/mL, *p* = 0.03). Despite their comparable EPA+DHA content, short-term consumption of salmon but not YSS induced significant changes in lipid profile and inflammatory markers. Larger clinical trials are needed to confirm the findings.

## 1. Introduction

Malaysia is among the world’s biggest consumers of fish, eating at least 56.5 kg of fish per person each year [1]. More than three quarters of Malaysians consume fish at least twice per week, eating 168 g of fish per day [2]. Eating fish on a regular basis can be beneficial for our health in many ways. For example, fish could help to reduce the risk of cardiovascular disease (CVD) and improve the chances of survival following a heart attack, as displayed in several observational studies [3,4,5], although not all agree [6]. Researchers believe that the heart health benefit of fish is more promising in those fish rich in omega-3 fatty acids, which is attributed to the ability of fish to ameliorate hypertriglyceridemia [7,8] and resolve inflammatory processes [9]. A recent meta-analysis demonstrated that consuming oily fish was associated with significant reductions in plasma triglycerides and an increase in HDL-cholesterol [10]. Omega-3 fatty acids reduce plasma lipid levels by inhibiting triacylglycerol and VLDL synthesis in liver, increasing fatty acid oxidation, as well as promoting the synthesis of membrane phospholipids [11,12,13]. Increased dietary EPA intake appears to compete with arachidonic acid (AA, omega-6 fatty acids) for the same desaturation enzymes, and in turn produces eicosanoids that are less potent than those produced from AA [14]. Pro-resolving mediators such as resolvins and protectins derived from EPA and DHA also helps in regulating the inflammatory response [9]. Omega-3 fatty acids are essential polyunsaturated fatty acids that must obtain from diet due to the lack of delta-12 and delta-15 desaturases in humans [15]. Eicosapentaenoic acid (EPA) and docosahexaenoic acid (DHA) are the two main long-chain omega-3 fatty acids commonly found in marine sources [16]. Marine fish, especially salmon, are the principal source of EPA and DHA [17]. In Malaysia, people consume mostly farmed Atlantic salmon, particularly Norwegian salmon [18]. However, salmon can be costly and require importation for tropical countries such as Malaysia. Yellowstripe scad (YSS, *ikan selar kuning*) is one of the most frequently consumed local fish in Malaysia [2]. Many Asian populations like to consume it as snack in the form of dried fish while Malaysians usually deep-fry and serve it with nasi lemak. It is not only commonly available in Malaysia but also affordable in price. Recent studies showed that YSS could provide a comparable eicosapentaenoic acid and docosahexaenoic acid (EPA+DHA) content to farmed Atlantic salmon (879 mg/100 g vs. 947 mg/100 g) [19,20]. However, studies exploring the benefits of YSS are scarce. The current study previously demonstrated an alteration of leptin and prothrombotic parameter upon the consumption of YSS and salmon [21]. In this paper, we aimed to investigate the effects of consuming YSS as compared with salmon on lipid profile and inflammatory markers among healthy overweight subjects.

## 2. Materials and Methods

### 2.1. Study Design and Subjects

A randomized, two-period crossover trial was carried out under free-living conditions among staff and students in Universiti Putra Malaysia (UPM) from October 2016 to May 2017. The Ethics Committee Research Involving Human Respondents of Universiti Putra Malaysia (JKEUPM) approved the study protocol. The trial was registered under the National Medical Research Register (NMRR-16-2693-3230) and ClinicalTrials.gov (NCT03251014). The subjects were healthy Malaysian adults aged 21–55 years and were overweight (BMI 23.0–27.4 kg/m²) according to the Asian BMI cut-offs [22]. Subjects were excluded if they reported (i) having cardiovascular disease, haemostasis disorder (haemophilia or thrombosis-related disorders), inflammatory disease, diabetes mellitus, hypertension (>140/90 mm Hg), or other significant medical history that could prohibit the participation; (ii) receiving warfarin/aspirin treatment, or medication to lower blood lipids, blood pressure, and inflammation; (iii) menopause, pregnancy, or lactating; (iv) consuming fish twice or more per week, or taking fish oil supplements for the last one month.

### 2.2. Diet

The intervention diets were steamed whole YSS (≈385 g whole fish/day, which corresponded to ≈265 g fillet/day) and farmed Atlantic salmon fillet (≈246 g fillet/day). Whole YSS was provided because it is too small in size, making it impractical to be filleted. An experiment on determining the net weight of YSS fillet was conducted by measuring the initial weight (whole fish) and final weight (fillet) of YSS after removing the head, bones, and tail. Based on the result, an additional weight of 45.1 % (30.7 ± 6.6 g) was taken into account when portioning whole YSS (total weight of 385 g/day). The amount of fish given was based on the EPA+DHA intake for greatest cardioprotection (1000 mg/day or 7000 mg/week) [23], as shown in Table 1. Subjects were required to consume the fish three times (days) per week during weekdays [24]. 

The fish was steamed in aluminium foil based on the method adopted from Koubaa et al. [25] with slight modification. The fish was seasoned with either salt or white pepper and covered with other ingredients (such as garlic, onion, ginger, chilies, and lemongrass) and sauces (such as soy sauce, oyster sauce, black pepper sauce, and so on). Water in the steamer was brought to boil before cooking the fish with the lid on for 20 min. Steaming was selected as the cooking method as it retains the most EPA and DHA content as compared with other cooking methods (baking in foil, frying, and grilling) [26].

### 2.3. Intervention

A randomized, two-period crossover trial was carried out under free-living conditions. All the subjects were randomly assigned to either the first or second group. A tamper-proof block randomization procedure was followed to ensure an equal allocation ratio. The random allocation sequence was created using computer-generated random numbers. Unlike other types of treatment such as diet pills, the diet treatment of this study could not be matched in taste, texture, and appearance, thus making the blinding of researchers and subjects impossible. To acknowledge the potential bias from a lack of blinding, precautions were made by ensuring both allocation groups were treated as equally as possible through the standardization of co-intervention, frequency of follow-up, and care of subjects [27]. 

The first group of subjects received YSS followed by salmon, while the second group received salmon followed by YSS. The diets were administered in the form of a lunchbox containing the steamed fish dishes served with one serving of cooked white rice (1 cup, cooked) and vegetable side dish (½ cup, cooked) [28]. All the foods were the same across the groups except for the types of fish. Subjects were instructed to consume one lunchbox in a day for three days per week. The same type of cooked fish was given for eight weeks, with a washout period of 8 weeks between the two interventions [29]. Throughout the intervention period, subjects were instructed to refrain from consuming omega-3 rich food entirely but otherwise to make no changes in their diets. A list of commonly consumed omega-3 rich food was provided to the subjects (Table 2). This is to minimize the bias toward overestimating the intervention effect [30]. Subjects were required to snap a photo of the lunchbox (including possible leftovers) after their meal to monitor their compliance to the diet given [31]. The compliance index was calculated based on the following formula [32]:

$$\mathrm{Compliance}\;\mathrm{index}\;(\%)=\frac{\mathrm{Total}\;\mathrm{number}\;\mathrm{of}\;\mathrm{treatment}\;\left(\mathrm{lunchbox}\right)\;\mathrm{consumed}}{\mathrm{Total}\;\mathrm{number}\;\mathrm{of}\;\mathrm{treatment}\;\left(\mathrm{lunchbox}\right)\;\mathrm{theorectically}\;\mathrm{required}}\times 100$$

Good compliance is defined as no more than a 10% deviation from absolute adherence [32]. All the subjects achieved at least 90% of the compliance index.

### 2.4. Outcome Measures

Body weight and height were assessed before and after each diet period. The compliance with dietary intake and physical activity was monitored through self-administered two-day 24-h dietary recall (24HR) [33] and International Physical Activity Questionnaires (IPAQ) [34], respectively. A fasting blood sample (7 mL) was taken to determine the serum EPA+DHA level, blood lipid profile, and inflammatory markers.

The serum EPA+DHA level was performed based on the modified method described elsewhere [35,36]. The samples were methylated using boron trifluoride (BF3)/methanol and analysed by gas chromatography. Gas chromatographic analysis was performed on an Agilent 6890 gas chromatograph equipped with flame ionization detectors, and a capillary column (DB-23, 0.25 mm × 60 m, 0.15 μm film, J & W Scientific) [37]. Fatty acid methyl esters were identified by comparing the retention times to those of known standards. Methyl heptadecanoate (C17:0) was used as an internal standard. 

The lipid profile was analysed by a medical diagnostic laboratory (Clinipath Malaysia Sdn Bhd, Selangor, Malaysia). In the medical diagnostic laboratory, the analysis of total cholesterol and triglycerides was performed on a fully automatic biochemistry analyzer (COBAS 6000 analyzer, Roche Diagnostics, Mannheim, Germany). Serum HDL-cholesterol was determined calorimetrically using the HDL separation tab (Union Carbide Corporation, Pleasantville, NY, USA). VLDL-cholesterol was estimated by dividing triglyceride level with a factor of 2.2, whereas LDL-cholesterol was calculated using the Friedewald formula [38]: 

LDL-cholesterol (mmol/1) 

= Total cholesterol (mmol/1) − HDL-cholesterol (mmol/1) − VLDL-cholesterol (mmol/1), 

where by VLDL-C (mmol/1) = TG (mmol/1)/2.2. 

The atherogenic coefficient (non-HDL-cholesterol/HDL-cholesterol) was determined, whereby non-HDL cholesterol was calculated by subtracting the HDL-cholesterol value from the total cholesterol. The atherogenic index of plasma (AIP) was calculated as log_10_ (TG/HDL-C) [39].

Inflammatory markers, including IL-1β, IL-6, TNF-α, and IFN-α, were determined using a custom Magnetic Luminex human premixed multi-analyte kit (R&D Systems, Minneapolis, MN, USA). As stated by the manufacturer, the detection limits for IL-1β, IL-6, TNF-α, and IFN-α were 0.11 pg/mL, 0.10 pg/mL, 0.10 pg/mL, and 0.09 pg/mL; whereas sensitivities of the assay were 0.27 pg/mL, 0.36 pg/mL, 0.60 pg/mL, and 0.31 pg/mL.

### 2.5. Statistical Analysis

The analyses presented were among the prespecified secondary outcomes of the present study, while thrombosis as the primary outcome was the basis for the sample size calculation [21]. The sample size was calculated using the two-sided comparison of means in a repeated measures design [40]. It was determined by designing the trial to have 90% power and an α of 0.05 to detect a significant difference. A power calculation was performed based on the documented difference of 2.32 % in plasma plasminogen activator inhibitor-1 upon DHA supplementation [41]. An effect size of 0.6925 [41] and an assumed attrition rate of 20 % indicated a minimum sample size of 48 subjects per group. 

All data obtained were analyzed using IBM SPSS Statistics version 23 (IBM Corp, Armonk, NY, USA). Exploratory data analysis was initially carried out to check for the normality of data. Any skewed variables were normalized by natural log transformation. If the variables remained skew after data transformation, non-parametric tests were applied. Analyses followed the intention-to-treat principle, categorizing participants according to the randomized assignment of treatment regardless of compliance. The differences between baseline and end values for each diet group were tested using paired samples *t*-test for parametric data and Wilcoxon signed-rank test for non-parametric data. Two-way repeated measures analysis of variance (ANOVA) with baseline values as a covariate was used to determine the differences between YSS and salmon [42]. All *p*-values < 0.05 were regarded as significant.

## 3. Results

**Figure 1:**
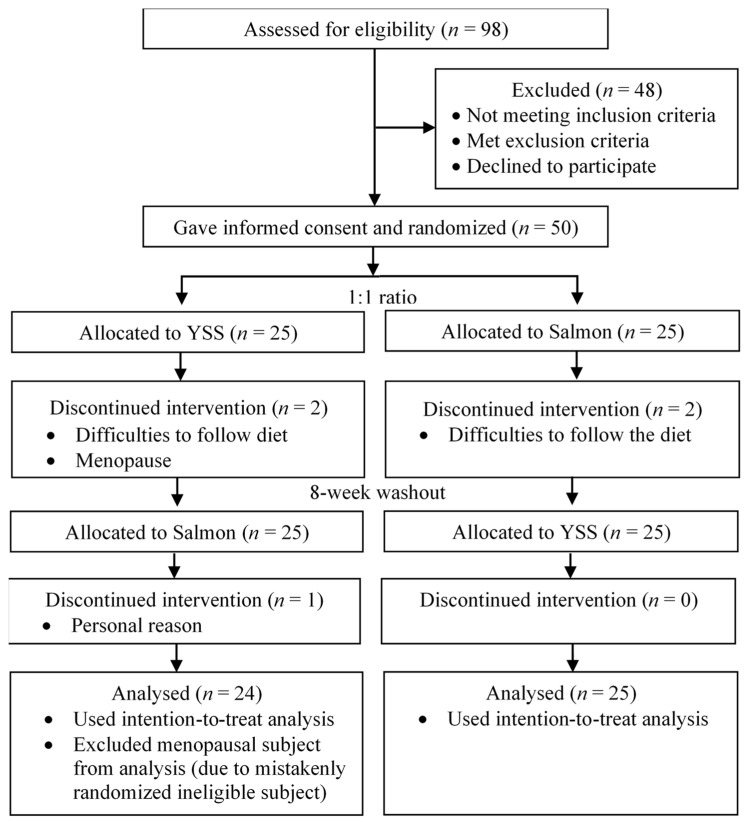
CONSORT subject flow diagram over the 24-week study period.

Of the 98 subjects screened, 50 subjects were recruited in the study. There was an 8% dropout rate due to the difficulty of following the diet (*n* = 3) and personal reasons (*n* = 1). One menopausal subject was retrospectively excluded because she was mistakenly randomized into the study but did not receive any intervention. The exclusion of data under this circumstance did not affect the results [43]. The final analysis included 49 subjects based on the intention-to-treat principle. Figure 1 outlines the flow of participants over the 24-week study period based on CONSORT 2010 statement. The socio-demographic characteristics of the subjects are summarized in Table 3.

During the intervention, three samples of respective cooked fish were randomly selected to analyse for their fatty acid content via gas chromatography analysis by an established medical diagnostic laboratory (Unipeq Sdn Bhd, Selangor, Malaysia). The fatty acid composition of fish is presented in Table 4. The comparison of EPA and DHA content between YSS and salmon is further discussed in Section 3.4.

Table 5 presents the changes in dietary intake, physical activity, serum EPA+DHA level, blood lipid profile, and inflammatory profile of subjects after 8-week consumption of YSS as compared with salmon. The baseline values of all the measured variables were comparable between two diet groups, indicating the absence of carry-over effects. The energy intake, macronutrient intake, and physical activity level of subjects in both groups remained unchanged throughout the intervention. The body mass index was unlikely to be different from baseline and across diet groups. The observed changes confirmed a good compliance of subjects to the study instructions. 

Only the salmon group demonstrated a threefold increase in EPA intake (*p* = 0.002) and twofold increase in DHA intake (*p* = 0.002). Pairwise comparison using Friedman’s tests revealed that the between-group difference was significant on EPA intake (median difference of −0.03 g/day, *p* = 0.01), but not on DHA intake (median difference of −0.03 g/day, *p* = 0.24). 

### 3.1. Serum EPA+DHA Level

There were no changes in serum EPA+DHA levels after the consumption of YSS and salmon (Table 5). When the omega-3 fatty acids were assessed individually, although salmon increased the serum EPA twofold (*p* = 0.04) at 8 weeks, the between-group difference was not significant. No significant changes were found on serum DHA with both diets. 

### 3.2. Blood Lipid Profile

YSS did not induce significant changes to the blood lipid profile. Conversely, salmon significantly reduced triglycerides by 15.1 % (between-group difference: −0.09 mmol/1, *p* = 0.01),VLDL-cholesterol by 14.6 % (between-group difference: −0.04 mmol/1, *p* = 0.01), and AIP by 0.4 % (between-group difference: −0.05 mmol/1, *p* = 0.006) as compared with YSS. The results suggest that the effect of salmon on reducing triglycerides, VLDL-cholesterol, and AIP was significantly stronger than YSS. HDL-cholesterol increased after the consumption of salmon (within-group difference: +3.9 %, *p* = 0.008), with no significant difference between the two diets (Table 5). 

### 3.3. Inflammatory Markers

YSS did not induce changes on the inflammatory markers IL-1β, IL-6, TNF-α, and IFN-γ at 8 weeks (Table 5). On the contrary, there was a significant reduction in IL-6 and TNF-α from baseline with salmon (within-group difference: −5.9 % and −2.2 %, *p* = 0.03 and <0.001). However, significant relative change between salmon as compared with YSS was only observed on IL-6 (between-group difference of log (IL-6): −0.01 pg/ml, *p* = 0.03) but not TNF-α. Other inflammatory markers did not change significantly with salmon at 8 weeks. 

### 3.4. EPA+DHA Content of Fish

As shown in Table 6, the mean EPA+DHA contents of YSS and salmon were 769.82 ± 80.48 mg/100 g sample and 1011.16 ± 94.40 mg/100 g sample, respectively. The current findings were comparable with those reported in the previous study [19,20]. Although the EPA+DHA content of YSS was lower than salmon, the difference was not significant. When EPA and DHA were assessed individually, YSS had significantly lower EPA content as compared with salmon (between-group difference: −0.03 g/day, *p* = 0.01), whereas the DHA content was similar between YSS and salmon. 

## 4. Discussion

The present study investigated the effect of YSS as compared with salmon on the lipid profile and inflammatory markers among healthy overweight subjects. A crossover design was selected for several reasons. Firstly, the intervention is evaluated within the same participants, thus eliminating the between-subject variability [44]. Secondly, this study design is more sensitive than parallel-group design to detect small differences between equivalent treatments such as YSS and salmon that have similar EPA+DHA content [45]. Thirdly, since each participant serves as his or her own control, fewer subjects are required to achieve a similar statistical power [46]. The 8-week duration of the diet period was chosen as this time frame was sufficient to incorporate EPA+DHA into tissues [47] and to induce notable effects in the lipid profile [8] and inflammation [48]. A washout period of 8 weeks allowed to reduce the possible carry-over effect from the crossover study design, as indicated by the insignificant changes between the baseline values of YSS and salmon.

The consumption of YSS did not induce significant benefits on the lipid profile. Instead, salmon improved triglycerides and VLDL-cholesterol levels. Published interventional data displaying the lipid-lowering effects of dietary fish are conflicting. A crossover trial by Lindqvist et al. [49] documented that the triglyceride-lowering effect by 6-week consumption of herring fish (equivalent to 1200 mg EPA+DHA/day) was no difference from pork and chicken fillets (equivalent to 400 mg EPA+DHA/day). The author concluded that total dietary composition (protein, carbohydrates, and fat) rather than increased fish intake plays a crucial role in improving the triglyceride level. Unfortunately, a later study did not support this hypothesis [8]. The 8-week, parallel-arm, randomized intervention study demonstrated a dose-dependent relationship between the triglyceride-lowering effect of fatty fish diets and their EPA+DHA intake, suggesting that dietary EPA+DHA intake played a higher role than total dietary composition [8]. In contrast, the current results reported a significant triglyceride-lowering effect by salmon compared with YSS, although both dietary fish had similar EPA+DHA content. With that, whether total dietary composition or high omega-3 fish diet contribute to the lowering effect on triglycerides is far less certain. 

The atherogenic index of plasma (AIP) is the most sensitive marker for predicting cardiovascular risk compared with other atherogenic indices, including atherogenic coefficient [50]. AIP values increase with the cardiovascular risk [39]. In this study, AIP reduced significantly after the consumption of salmon as compared with YSS, indicating that salmon is superior in improving the lipid profile and the occurrence of cardiovascular events. Derosa et al. [51] found an increase in HDL-cholesterol and decreased triglycerides after 18 months of omega-3 supplementation. Although consuming fresh fish seems to provide more promising benefits in AIP than omega-3 supplement [52], research exploring the effect dietary fish on AIP is still scarce. Devadawson et al. [53] showed a statistical difference between inland fish and sea fish eaters on the AIP level; however, which group of fish eaters had a better profile was not stated clearly.

Moreover, HDL-cholesterol was significantly increased by 3.9% or 0.06 mmol/1 from baseline with the consumption of salmon. According to Gordon et al. [54], each increase in baseline HDL-cholesterol of 0.03 mmol/1 is associated with a 6% decrease in the risk of death from CVD. In this case, the improvement of salmon on HDL-cholesterol presented in the current study would be unequivocally of great benefit to the population. Previously, LDL-cholesterol was significantly reduced following 8-week consumption of Namibia hake, a type of white fish rich in omega-3 fatty acids (equivalent to 642 mg EPA+DHA/day) [55]. The current study findings, however, are not in line with the previous evidence. Being also a type of white fish rich in omega-3 fatty acid, the consumption of YSS (equivalent to 1000 mg EPA+DHA/day) did not exert significant effect LDL-cholesterol. The study by Vazquez et al. [55] explained that the observed reduction in serum LDL-cholesterol may not be a direct effect of omega-3 fatty acid content in fish; instead, a reduced intake of saturated fats from other protein sources may lead to lower LDL-cholesterol among subjects during the period of fish intake. These results are in keeping with the hypothesis from the previous study that reduced saturated fat intake rather than omega-3 content of fish contributed to a lower level of LDL-cholesterol. Future trials are warranted to confirm this hypothesis. 

Despite the beneficial difference in lipid profile observed in the salmon group, it should be noted that the mean baseline lipid levels in both diet groups were within the normal range or at borderline. According to the Clinical Practice Guideline of Malaysia, a person is diagnosed with dyslipidaemia when he/she has a total cholesterol of >5.2 mmol/l, triglycerides of >1.7 mmol/l, HDL-cholesterol of <1.0 mmol/l (males) or <1.2 mmol/l (females), and/or LDL-cholesterol of <3.0 mmol/l or <3.8 mmol/l (if triglycerides >4.5 mmol/l) [56]. Although the observed improvement in the salmon group may not be clinically meaningful to the study population, it may at least be useful as primary prevention. Moreover, it is possible that other dietary nutrients and physical activity levels could have influenced the results, although both diet groups did not show significant differences in terms of diet quality [57,58]. The increasing trend of carbohydrate intake and reducing trend of physical activity level observed in the YSS group after 8 weeks were much greater than the salmon group, though not statistically significant. 

In terms of inflammatory markers, current results demonstrated no significant changes with the consumption of YSS. On the contrary, salmon was found to exert a significant suppression effect on the pro-inflammatory marker IL-6 compared with YSS. Serum TNF-α was also decreased significantly in the salmon group after 8 weeks but not significantly in between-group difference. Results of the present study corroborate those reported by Zhang et al. [8] regarding the beneficial effect of 8-week salmon consumption on lowering and IL-6 and TNF-α levels. Conversely, a later randomized trial using mixed fish, equivalent to approximately 800 mg/day of EPA+DHA, did not support the beneficial effect of consuming omega-3 rich fish for 8 weeks on serum cytokines levels [44]. It is believed that the dietary EPA+DHA intake in Grieger et al. [59] (800 mg/day) was lower than that in Zhang et al. [8] (1600 mg/day), and the present study (1000 mg/day) to improve the inflammatory markers. 

There are several possible explanations for the varied effects of YSS and salmon on health outcomes. First, dietary inclusion of YSS and salmon did not effectively increase serum EPA+DHA despite their similar omega-3 content. When the omega-3 fatty acids were studied individually, higher EPA intake reflected from salmon, which had higher EPA content, increased the serum EPA level of subjects after 8 weeks. There is growing evidence that EPA and DHA exert differential health benefits [60,61]. The current findings acknowledge that the observed changes may mainly be associated with dietary intake of EPA. However, research remains inconclusive as to whether EPA or DHA is better to improve outcomes [60,61]. Considering the potentially independent effects of EPA or DHA on outcomes would be meaningful in the future. Second, YSS and salmon have different omega-6 to omega-3 fatty acids (n6:n3) ratio. A recent study found that even with similar EPA+DHA content, the beneficial effects on lipid profile and inflammatory markers tend to be greater when consumed fish with a lower ratio of omega-6 to omega-3 fatty acids (n6:n3 ratio) [62]. If this effect is confirmed, it could explain the significant changes in lipid profile and inflammatory markers observed in the salmon group instead of the YSS group since salmon had a lower n6:n3 ratio than YSS (0.3 vs. 0.2) [19,20]. Taken together, we can speculate that higher dietary EPA intake and lower n6:n3 ratio may be associated with greater beneficial effects on the lipid profile and inflammatory markers. Further studies are necessary to confirm this hypothesis.

As discussed in our previous study [63], the amount of fish given was nearly double the recommended serving size (150 g) [64], although the intention was to mimic the omega-3 intake recommendation (1000 mg/day or 7000 mg/week) [23]. Consuming such an amount of fish is unlikely to be practical in the long term. Harris et al. [65] observed that incorporating EPA+DHA into blood lipids was equally effective when provided an identical amount of EPA+DHA from oily fish on a weekly basis or from fish oil supplementation on a daily basis. However, an in vitro study carried out by Browning et al. [66] reported to have better EPA+DHA status when consuming a moderate amount regularly (daily) instead of a higher amount intermittently (twice per week), despite both administrations providing an identical amount of EPA+DHA per week. A recent in vivo study suggested that having a large dose of omega-3 fatty acids once per week is more effective than a smaller dose delivered daily [67]. Additional research to compare the effectiveness of taking EPA+DHA derived from oily fish consumption on a daily and intermittently basis is warranted. 

To our best knowledge, this is the first randomised crossover study that compared the health benefits of YSS and salmon matched for EPA+DHA content. This study design is one of the most powerful designs for examining the efficacy of dietary treatments [68]. However, there are several limitations that should be addressed in this study. First, the dietary data and the serum EPA+DHA compliance biomarker showed no increase in the YSS period and very little increase in the salmon period, despite a goal of reaching an intake of approximately 1000 mg/day EPA+DHA. The results raise doubts about whether the subjects truly adhered to the study protocol, although subjects reported a good compliance index based on meal photos taken. Poor compliance is not uncommon in randomized trials with free-living subjects. One of the possible reasons for the non-compliance in this study is that the fish was given at nearly twice the recommended serving size, which was not usual. Therefore, subjects may fail to finish the diet treatment. Moreover, YSS is rather small and bony and is commonly prepared by deep-frying. Instead, the fish was steamed to retain most of its nutrients in this study. This little bony fish may cause hassle to the eating process. Moreover, salmon is a favourite fish of people but is also expensive; not all could afford it. It cannot rule out the possibility that subjects may share the diet treatment with family or friends. All these factors may relate to subject’s non-compliance in this study [69,70]. The primary purpose of self-administered two-day 24HR was to monitor the compliance with dietary intake. Although the intake of other nutrients was generally not different between groups, we did not specify which two days were to be recorded. This presents a further limitation to the study. 

Second, in the earlier work of this study, which reported preliminary findings for the first intervention period [42], the YSS group had higher total cholesterol and HDL-cholesterol after 8 weeks [42]. Nevertheless, the modulation of YSS on these outcomes did not last on the second intervention period. A possible explanation is that subjects’ compliance was compromised due to the duration of intervention and complexity of the diet treatment [69]. At the second intervention phase, subjects in the second group may have less motivation since it was more hassle to consume the bony fish YSS than salmon. 

Third, even though there were statistical differences of decreasing IL-6 and TNF-α after 8 weeks of salmon consumption and between-group difference on IL-6 concentration, the changes were very small. Therefore, it is difficult to conclude the beneficial effects of salmon on these biomarkers. 

Forth, the sample size was calculated based on the effect size of the primary outcome while the variables discussed in this paper were the secondary outcomes. This study had relatively wide confidence intervals that crossed zero for most variables. This may indicate an inadequate sample size and the study may not have adequate power to detect existing differences. We considered that this study would produce possible differences in the study outcomes due to results demonstrated by previous clinical trials of omega-3 rich fish [8,23,54,55,59]. Therefore, more extensive human clinical trials with larger sample sizes are necessary.

## 5. Conclusions

We concluded that short-term consumption of YSS and salmon at a dosage equivalent to approximately 1000 mg/day of EPA+DHA did not exert similar effects on lipid profile and inflammatory markers among healthy overweight adults. Salmon with a higher EPA content elicited greater significant health benefits than YSS.

## Figures and Tables

**Table 1:** Amount of fish given.

Diet	Amount Given	Frequency	Corresponding EPA+DHA Content *
Steamed YSS	≈385 g whole fish/day **	3 times (days)/week	2329 mg/day (≈7000 mg/week)
Steamed salmon	≈246 g fillet/day	3 times (days)/week	2330 mg/day (≈7000 mg/week)

* Based on 879 mg EPA+DHA/100 g fillet of YSS [13] and 947 mg EPA+DHA/100 g fillet of farmed Atlantic salmon [12]. ** Correspond to ≈ 265 g fillet/day.

**Table 2:** List of omega-3 rich food.

Food Group	Food
Fish and seafood	Salmon
	Yellowstripe scad
	Mackerel
	Sardine
	Tuna
	Trout
	Herring
	Threadfin bream
	Anchovies
Meat, eggs and poultry	Omega-3 enriched eggs
Dairy products	Omega-3 enriched milk
Nuts and seeds	Flaxseeds/linseeds
	Chia seeds
	Walnuts
Fats and oils	Cod liver oil
	Flaxseed oil
	Omega-3 enriched margarine

**Table 3:** Socio-demographic characteristics of subjects (*n* = 49).

Socio-Demographic Characteristics	*n*	%
Age (years)		
Mean	29	
SD	7	
Range	21–46	
Sex		
Male	17	34.7
Female	32	65.3
Ethnicity		
Malay	35	71.4
Chinese	12	24.5
Indian	2	4.1

**Table 4:** Fatty acid composition of fish.

Fatty Acid Composition	YSS	Salmon
Total fat (g/100g)	3.33	15.17
Saturated fat (mg/100 g sample)	1751.64	2557.48
Caprylic acid 8:0	1.61	0.66
Capric acid 10:0	0.00	2.38
Undecanoic acid 11:0	0.76	0.24
Lauric acid 12:0	26.29	169.65
Tridecanoic acid 13:0	2.46	1.96
Myristic acid 14:0	246.37	334.74
Pentadecanoic acid 15:0	58.94	30.36
Palmitic acid 16:0	895.24	1462.68
Heptadecanoic acid 17:0	25.97	35.65
Stearic acid 18:0	375.17	405.72
Arachidic acid 20:0	24.45	53.39
Henicosanoic acid 21:0	7.32	4.44
Behenic acid 22:0	21.26	27.80
Tricosanoic acid 23:0	55.26	19.84
Lignoceric acid 24:0	10.58	7.98
Monounsaturated fat (mg/100 g sample)	561.46	8017.96
Myristoleic acid 14:1	1.42	6.05
Cis-10-pentadecenoic acid 15:1	0.13	3.42
Palmitoleic acid 16:1	254.24	374.85
Cis-10-heptadecanoic acid 17:1	63.41	32.69
Elaidic acid 18:1n9t	3.60	0.00
Oleic acid 18:1n9c	210.96	6870.41
Cis-11-eicosenoic acid 20:1n9	5.48	324.02
Erucic acid 22:1n9	4.21	350.91
Nervonic acid 24:1	18.02	55.61
Polyunsaturated fat (mg/100 g sample)	1020.83	4593.94
Linolelaidic acid 18:2n6t	0.00	17.04
Linoleic acid 18:2n6c	73.65	2274.80
γ-Linolenic acid 18:3n6	13.19	40.29
α-Linolenic acid 18:3n3	28.52	894.95
Cis-11,14-eicosadienoic acid 20:2n6	14.54	173.17
Cis-8,11,14-eicosatrienoic acid 20:3n6	7.57	48.79
Cis-11,14,17-eicosatrienoic acid 20:3n3	99.92	40.15
Arachidonic acid 20:4n6	3.49	74.86
Cis-5,8,11,14,17-eicosapentaenoic acid 20:5n3	214.16	400.52
Docosadienoic acid 22:2	10.16	18.75
Cis-4,7,10,13,16,19-docosahexaenoic acid 22:6n3	555.66	610.63

**Table 5:** Changes in dietary intake, physical activity, serum EPA+DHA level, blood lipid profile, and inflammatory profile of subjects after intervention (*n* = 49).

	YSS			Salmon			Between−Group Difference ^2^	95% CI	*p* Value **
	Baseline ^1^	Week 8	*p* Value *	Baseline ^1^	Week 8	*p* Value *			
Dietary intake									
Energy (kcal/day)	1359 (368)	1448 (487)	0.16	1356 (357)	1413 (548)	0.44	32.00	−135; 206	0.06
Carbohydrate (g/day)	58.61 (23.82)	68.29 (25.66)	0.06	58.79 (20.63)	60.76 (27.11)	0.49	7.71	−1.64; 16.71	0.18
Protein (g/day)	173.78 (52.23)	181.01 (68.33)	0.42	173.47 (45.44)	180.08 (65.52)	0.44	0.62	−19.78; 21.63	0.10
Total Fat (g/day)	47.61 (18.75)	50.28 (26.37)	0.31	47.53 (17.73)	49.93 (25.82)	0.49	0.27	−7.59; 8.30	0.10
PUFA 20:5, EPA (g/day) ^3^	0.01 (0.04)	0.00 (0.02)	0.34 ^§^	0.01 (0.03)	0.03 (0.15)	0.002 ^§^	−0.03	0.00; 0.05	0.01 ^†^
PUFA 22:6, DHA (g/day) ^3^	0.04 (0.11)	0.04 (0.11)	0.74 ^§^	0.03 (0.06)	0.06 (0.20)	0.002 ^§^	−0.03	0.01; 0.15	0.24 ^†^
Saturated fat (g/day)	13.66 (6.84)	11.34 (6.51)	0.66	14.76 (10.56)	13.40 (10.87)	0.37	−0.96	1.55; 5.67	0.71
Total PA (MET-min/wk)	4134 (3571)	3909 (4598)	0.09	5326 (6298)	5157 (5273)	0.55	−56	−2826; 331	0.24
Body mass index (kg/m^2^)	25.20 (1.57)	25.14 (1.69)	0.25	25.29 (1.59)	25.27 (1.66)	0.78	−0.040	−0.28; 0.03	0.48
Serum EPA+DHA (ug/mL)	94.31 (150.56)	100.46 (90.34)	0.81	98.21 (78.99)	123.97 (116.48)	0.11	−19.61	−45.95; 14.04	0.29
Serum EPA	3.80 (16.02)	2.05 (10.06)	0.43	4.47 (13.75)	13.75 (26.62)	0.04	−11.03	−8.63; 1.10	0.13
Serum DHA	90.51 (150.07)	98.41 (89.65)	0.76	93.73 (78.91)	110.22 (114.85)	0.32	−8.59	−41.73; 17.34	0.41
Blood lipid profile									
Total cholesterol (mmol/1)	5.18 (0.83)	5.19 (0.97)	0.71	5.12 (0.15)	5.24 (0.89)	0.14	−0.11	−0.24; 0.12	0.92
Triglycerides (mmol/1)	1.11 (0.52)	1.04 (0.47)	0.38	1.06 (0.49)	0.90 (0.38)	<0.001	0.09	0.06; 0.22	0.01
HDL-cholesterol (mmol/1)	1.56 (0.27)	1.55 (0.30)	0.88	1.56 (0.37)	1.62 (0.29)	0.008	−0.07	−0.13; −0.01	0.41
LDL-cholesterol (mmol/1)	3.12 (0.77)	3.11 (0.85)	0.86	3.08 (0.88)	3.19 (0.82)	0.13	-0.12	−0.23; 0.08	0.20
VLDL-cholesterol (mmol/1)	0.51 (0.23)	0.48 (0.22)	0.55	0.48 (0.22)	0.41 (0.17)	<0.001	0.04	0.03; 0.11	0.01
Atherogenic coefficient	2.43 (0.76)	2.44 (0.88)	0.79	2.39 (0.86)	2.34 (0.83)	0.43	0.06	−0.04; 0.18	0.19
AIP	−0.18 (0.22)	−0.21 (0.22)	0.31	−0.20 (0.24)	−0.28 (0.20)	<0.001	0.05	0.01; 0.08	0.006
Inflammatory markers (pg/ml) ^4^									
IL-1β	1.17 (0.16)	1.14 (0.16)	0.77	1.13 (0.15)	1.13 (0.16)	0.19	−0.03	−0.02; 0.05	0.97
IL-6	0.69 (0.21)	0.66 (0.21)	0.43	0.68 (0.18)	0.64 (0.20)	0.03	0.01	−0.04; 0.07	0.03
TNF-α	1.38 (0.19)	1.36 (0.21)	0.19	1.37 (0.21)	1.34 (0.21)	0.007	0.01	−0.01; 0.04	0.95
IFN-γ	2.15 (0.36)	2.12 (0.36)	0.91	2.10 (0.33)	2.11 (0.35)	0.43	−0.04	−0.03; 0.05	0.25

All values are expressed as mean (SD), unless otherwise stated. PUFA: polyunsaturated fatty acid; EPA: eicosapentaenoic acid; DHA: docosahexaenoic acid; PA: physical activity; HDL: high-density lipoprotein; LDL: low-density lipoprotein; VLDL: very low-density lipoprotein; AIP: atherogenic index of plasma; IL: interleukin; TNF: tumour necrosis factor; IFN: interferon. ^1^ No significant differences were found on baseline values between diet groups for all the variables. ^2^ With respect to salmon group. ^3^ Values as median (IQR). ^4^ Values from natural log transformation. * Differences from baseline by paired-samples *t*-test. ^§^ Differences from baseline by Wilcoxon signed-rank test. ** Differences between diet groups by two-way repeated measures ANOVA adjusted for sex, age, ethnicity, and baseline values. ^†^ Differences between diet groups by Friedman ANOVA by ranks.

**Table 6:** EPA+DHA content of YSS as compared with salmon (*n* = 3).

Nutrient Content (mg/100 g Sample)	YSS	Salmon	Between-Group Difference ^§^	95% CI	*p* Value
Total EPA+DHA	769.82 (80.48)	1011.16 (94.40)	−241.35	−627.95; 145.26	0.11
EPA	214.16 (22.77)	400.53 (17.32)	−186.37	−273.40; −99.33	0.01
DHA	555.66 (57.71)	610.64 (77.08)	−54.98	−347.93; 237.97	0.50

All values are expressed as mean (SD). EPA: eicosapentaenoic acid; DHA: docosahexaenoic acid. ^§^ With respect to salmon group.

## Data Availability

The data presented in this study are available on request from the first or corresponding author.
